# Biocompatibility of variable thicknesses of a novel directly printed aligner in orthodontics

**DOI:** 10.1038/s41598-025-85359-7

**Published:** 2025-01-25

**Authors:** Maximilian Bleilöb, Claudia Welte-Jzyk, Vanessa Knode, Björn Ludwig, Christina Erbe

**Affiliations:** 1 Department of Orthodontics and Dentofacial Orthopedics, University Medical Center of the Johannes Gutenberg-University Mainz, Augustusplatz 2, 55131 Mainz, Germany; 2 Department of Orthodontics, University of Homburg, Saar, Germany; 3 Private Practice of Orthodontics, Am Bahnhof 54, 56841 Traben-Trarbach, Germany

**Keywords:** Digital orthodontics, 3D printed aligners, 3D printing, Clear aligner, Aligner therapy, Direct printed aligners, Biocompatibility, Cytotoxicity, Resin, Cell biology, Health care, Medical research, Materials science, Materials for devices

## Abstract

Direct printed aligners (DPAs) offer benefits like the ability to vary layer thickness within a single DPA and to 3D print custom-made removable orthodontic appliances. The biocompatibility of appliances made from Tera Harz TA-28 (Graphy Inc., Seoul, South Korea) depends on strict adherence to a standardized production and post-production protocol, including UV curing. Our aim was to evaluate whether design modifications that increase layer thickness require a longer UV curing time to ensure biocompatibility. Specimens with varying layer thickness were printed to high accuracy using Tera Harz TA-28 and the Asiga MAX 3D printer (Asiga SPS ™ technology, Sydney, Australia). UV curing durations were set at 20, 30 and 60 min. Cytotoxicity was evaluated using the AlamarBlue assay on human gingival fibroblasts. Cell viability decreased with increasing specimen thickness (significant for 2 mm [*p* < 0.001], 4 mm [*p* < 0.0001], and 6 mm [*p* < 0.01]) under the manufacturer-recommended 20-min UV curing. Extending the curing time did not improve cell viability. However, cell viability never decreased by more than 30%, meeting EN ISO 10993-5 standards for non-cytotoxicity. The standard 20-minute UV curing protocol ensures the biocompatibility and patient safety of Tera Harz TA-28 for material thicknesses up to 6 mm.

## Introduction

Clear aligner therapy has become an increasingly popular treatment method among orthodontists as well as patients seeking orthodontic care worldwide^1^. The growing demand has led to constant improvements in materials and technologies offered by a rising number of different companies^2^. Consequently, the range of applications has expanded from simple cases, such as relapses and crowding, to more complex treatments in contemporary orthodontics^1^. Furthermore, in modern society, patients of all age groups ask for highly aesthetic, comfortable and more hygienic treatments – all of which is provided by clear aligner therapy^1,3–6^.

In recent years, major technological innovations in 3D printing, especially related to computer-aided design, biomaterials and manufacturing techniques, have enabled the production of in-office direct to print aligners (DPAs) – representing an innovation compared to the traditional thermoformed aligners^2,7^. DPAs made from Graphy’s resin Tera Harz TC-85 (Graphy Inc., Seoul, South Korea) have received CE and KFDA certification as well as FDA approval^8^. However, it is crucial to consider that this certification only applies to the finished DPAs, produced in strict adherence to the established production protocol, not to the product itself. After printing the DPAs have to undergo highly delicate post-processing involving centrifugation, UV light curing in a nitrogen atmosphere, washing in boiling water as well as drying^8^. Any disruption to this process can lead to reduced biocompatibility, resulting in adverse effects on patient safety, for which the working orthodontist is always responsible – also legally.

Recently, Graphy has launched an updated and modified version of its resin, Tera Harz TA-28 (Graphy Inc., Seoul, South Korea). While TA-28 and TC-85 share many of their components and physical properties, the modifications may result in differences in the DPAs biomechanical properties, performance and biocompatibilitiy^8^. However, this still needs to be confirmed by future research. TA-28 aligner resin, similar to other photopolymers, consists of epoxy resins, which in liquid form are highly toxic denaturing and inactivating proteins in cells^9^. Thus, similar to Tera Harz TC-85, post-processing still represents the crucial part of the manufacturing process and has to be conducted according to the same protocols.

DPAs made from Graphy’s resins herald a new era in clear aligner therapy offering a number of biomechanical advantages compared to their thermoformed counterparts^10–15^. The ability to increase layer thickness in specific areas improves the efficiency of prescribed tooth movements while reducing side effects^10^. The capability to manufacture customized appliances, such as the Twin-Block, regarded as another key advantage, also involves increased material thickness.

Despite these wooed benefits, it is currently unknown if such adaptations to the common clear aligner thickness of 0.5–1.5 mm require an altered post-processing protocol^16^. In particular, it is unknown if the duration of polymerization using UV light needs to be extended to still guarantee biological safe use in patients.

Although the increased popularity of Graphy’s resin in recent years has led to extensive reasearch on the biomechanical properties, biocompatibility and intraoral ageing processes of DPAs made from Tera Harz TC-85 (Graphy Inc, Seoul, South Korea), to date, no studies have evaluated the influence of design changes involving increased material thickness on the DPAs’ biocompatibility and cytotoxicity^17–20^.

Thus, manufacturing and using customized DPAs or functional appliances according to the patient’s individual needs always bears the risk of exposing cytotoxic materials. Considering previous mucosa irritation in multiple patients, further biocompatibility assessment is of utmost importance and clinical relevance^19^.

Therefore, our objective was to evaluate the biocompatibility and cytotoxicity of Graphy’s new Tera Harz TA-28 aligner resin and to determine whether design adaptations, which require increases in material thickness, result in any reductions in biocompatibility when post-processing is not adapted appropriately.

To address this aim, we tested the following null hypotheses:

1. The extension of the duration of UV curing during post-processing does not affect the biocompatibility of DPAs made from Tera Harz TA-28 (Graphy Inc., Seoul, South Korea).
2. The increase in layer thickness of DPAs made from Tera Harz TA-28 (Graphy Inc., Seoul, South Korea) does not require prolonged UV curing during post-processing to maintain biocompatibility.
3. Digitally designed specimens, made from Tera Harz TA-28 (Graphy Inc., Seoul, South Korea), can be 3D printed to a high degree of accuracy, regardless of modifications in UV curing duration or material thickness.

## Methods

### Digital design, 3D-printing and post-processing of the specimens

Circular specimens with a diameter of 1 cm and varying thicknesses of 0.5, 1, 2, 4 and 6 mm were digitally designed using the free web app Tinkercad (Autodesk, San Francisco, CA, United States). The STL files were exported to the Asiga Composer Software (Asiga SPS ™ technology, Sydney, Australia), where the specimens were orientated at a 90-degree-angle on the build platform. Support structures with a length of 2 mm and a diameter of 1 mm as well as a mesh covering the build plate were added. The specimens were printed using Graphy’s Tera Harz TA-28 aligner resin (Graphy Inc., Seoul, South Korea) and the Asiga MAX 3D printer (Asiga SPS ™ technology, Sydney, Australia). After printing, the aligners, along with their support structures and mesh, were carefully removed from the build platform. The specimens were placed in the Graphy Tera Harz Spinner V2 (Graphy Inc., Seoul, South Korea) to use centrifugal forces to remove any excess resin from their surface (500 rpm for 10 min). After spinning, the mesh and supports were removed manually. The specimens were cured for 20, 30 or 60 min at Level 2 in the Graphy Cure THC 2 (Graphy Inc., Seoul, South Korea) in a 95% nitrogen atmosphere before being washed in boiling water for 2 min. The thickness of all specimens was measured manually using a digital caliper gauge.

### Cell culture

Commercially acquired human gingival fibroblasts (HGFs, CLS Cell Lines Service GmbH, Eppelheim, Germany) were cultured in Dulbecco’s modified Eagle medium (DMEM; Thermo Fisher Scientific, Carlsbad, CA, USA) containing 4.5 g/L glucose, 10% fetal bovine serum (Thermo Fisher Scientific, Carlsbad, CA, USA), 100 U/ml penicillin, 100 µg/ml streptomycin and 50 mg/L L-ascorbic acid (Sigma Aldrich, Steinheim, Germany) at 37 °C, 5% CO_2_ and 95% humidity. For all experiments, passage four to ten were used for experimental setups. Accutase^®^ (Sigma–Aldrich Chemie GmbH, Steinheim, Germany) was used for cell detachment. The cells were counted and seeded at a density of 2000–3000 cells per well in 96 well culture plates (Greiner Bio-One, Frickenhausen, Germany) with 100 µl medium per well. The cells were allowed to adhere to the cell bottom for 24 h.

### Specimen testing

**Fig. 1:**
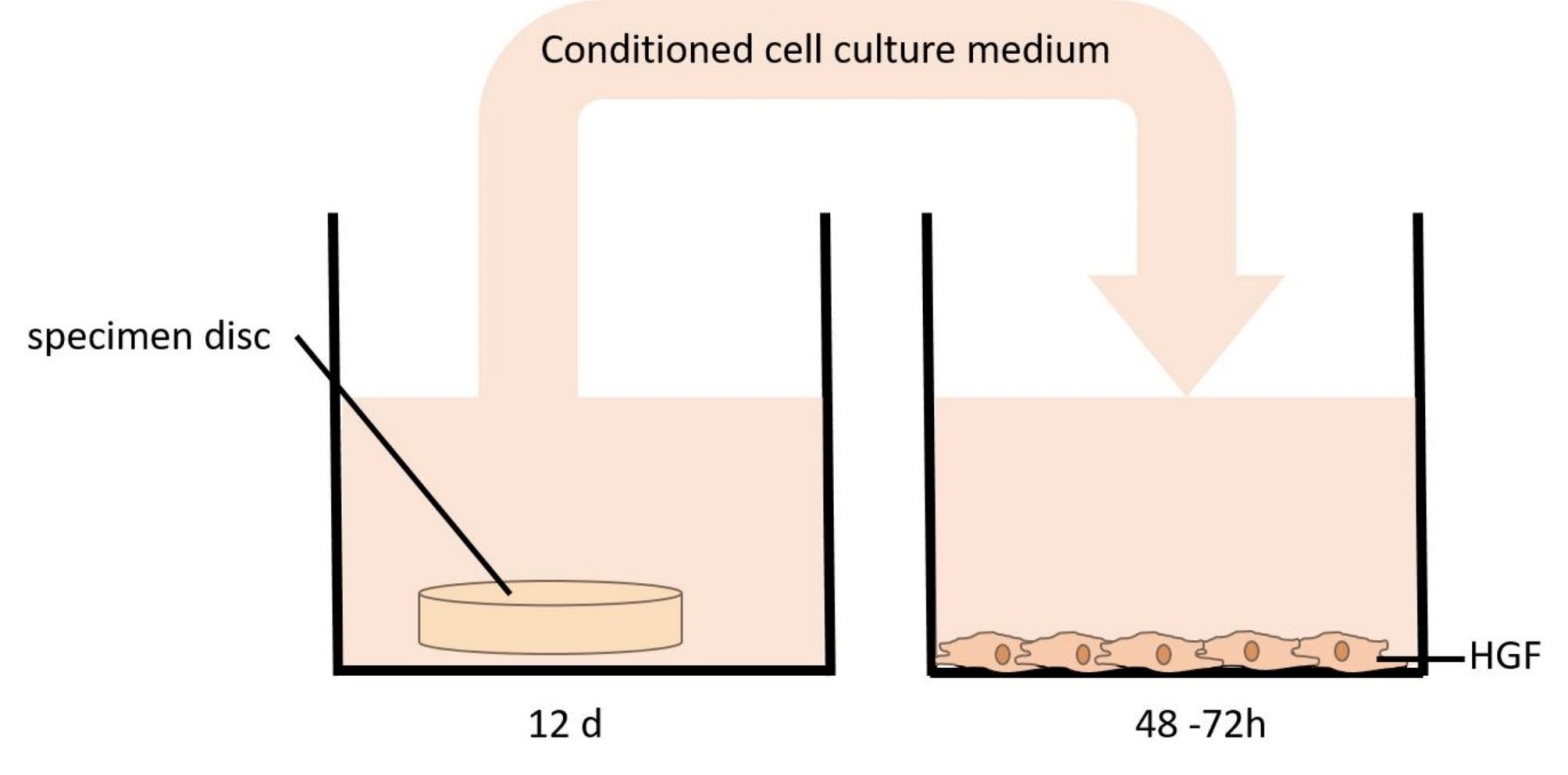
Specimen testing to evaluate the influence of the aligner material on human gingival fibroblasts (HGF).

The specimens were incubated in culture medium with intermittent shaking for 12 days, simulating the wear duration of clear aligners during active orthodontic treatment. To assess the potential impact of saliva exposure on cytotoxicity, additional samples were incubated in saliva for the corresponding period, evaluating its influence on cell viability. Following the incubation period, the medium or saliva (*n* = 6 for each thickness) was transferred to a 96-well plate containing adhered HGFs (2000–3000 cells per well) (Fig. 1).

Cell proliferation was monitored continuously based on the increase in confluence observed with the life-cell imaging device Incucyte (Incucyte, Sartorius, Göttingen, Germany). In addition, cell viability was measured using the AlamarBlue cell viability assay (AlamarBlue; ThermoFisher Scientific, MA, USA) after 48–72 h, when confluence has reached approximately 80%.

### AlamarBlue cell viability assay

Cell viability of the HGFs was assessed quantitatively using a colorimetric assay (AlamarBlu; ThermoFisher Scientific, MA, USA) according to the manufacturer’s protocol. The AlamarBlue cell viability reagent (ThermoFisher Scientific, MA, USA) is a non-toxic, cell-permeable indigo dye (Resazurin) used for quantitative analysis of cell viability and proliferation. It quantifies the metabolic turnover of living cells based on their reducing power. Upon entering living cells, Resazurin is reduced to Resorufin, a red and highly fluorescent compound. Changes in viability are easily detected with a fluorescence plate reader (excitation at 537 nm and emission at 600 nm, VersaMax Microplate Reader; Molecular Devices, Sunnyvale, USA). The premixed AlamarBlue reagent was added to the media above the cells in a dilution of 1:10 (no washing or cell lysis steps are required) and after an incubation time of one to four hours fluorescence was detected. Each experiment was performed with six replicates (*n* = 6 for each thickness), analyzing each approach in duplicate. Fluorescence values obtained were normalized to cells treated with media incubated for 12 days without specimens (= negative controls set as 100%), summarized as means and statistically analyzed. As the reagent is non-toxic, the cells can be used for further investigations after the assay. A reduction in cell viability by more than 30% in quantitative evaluation is considered a cytotoxic effect (ISO 10993-5, Tests for in vitro cytotoxicity). In addition, the cells were monitored microscopically, and slight cytotoxicity was graded when more than 20% of the cells were round or loosely attached, with only slight growth inhibition evident. According to ISO 10993-5, quantitative evaluation of cytotoxicity is preferable and qualitative means are appropriate for screening purposes.

### Statistical analysis

To identify differences between groups, an unpaired t-test or one-way ANOVA followed by Tukey’s post hoc test was performed. The standard error of the mean was calculated using GraphPad PRISM (GraphPad Software, Boston, MA, United States).

## Results

### Accuracy of specimen

**Fig. 2:**
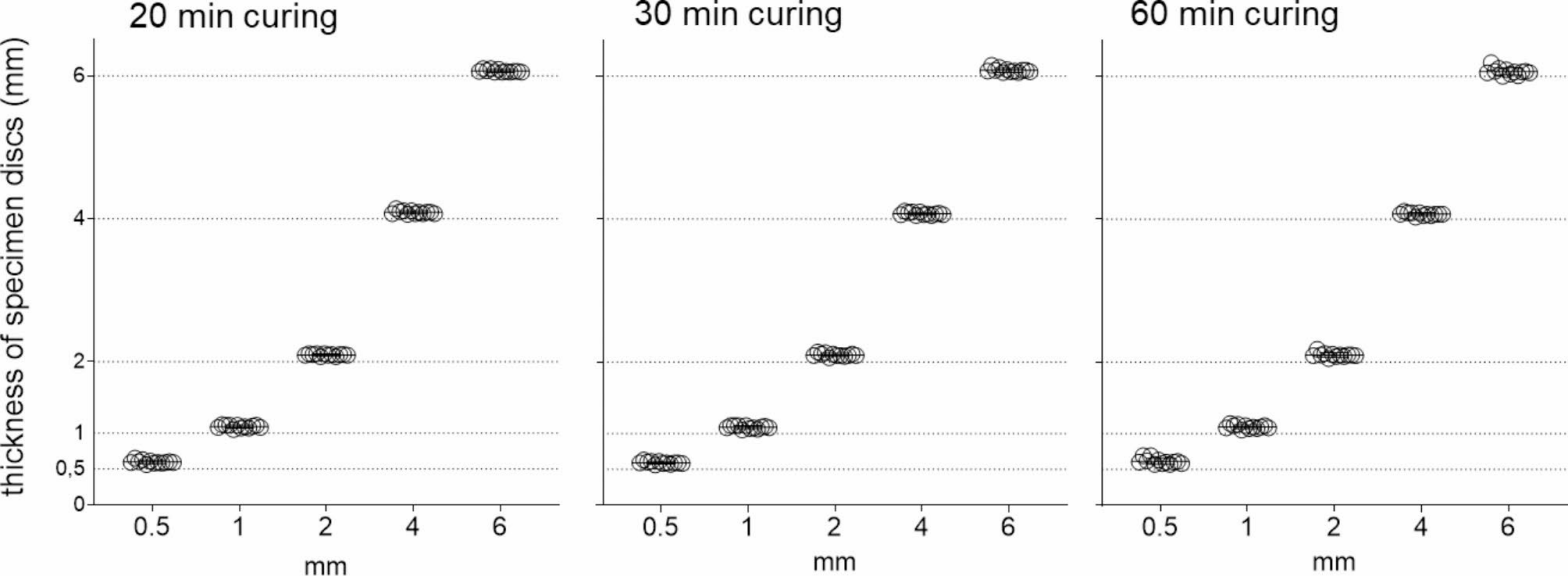
Measured thickness of specimens (*n* = 12) printed in different thicknesses (0.5, 1, 2, 4, 6 mm) using Graphy’s aligner resin Tera Harz TA-28 after UV curing with Tera Harz Cure for 20, 30 and 60 min respectively.

The specimens were manually measured using a digital caliper gauge and demonstrated high accuracy as they reached the desired diameter of 1 cm as well as various thicknesses of 0.5, 1, 2, 4, 6 mm. This precision was consistently maintained across all three different UV curing durations (Fig. 2).

### Cell proliferation and viability

**Fig. 3:**
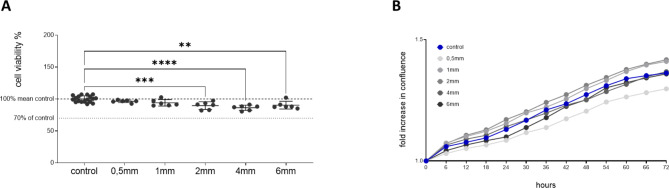
Cell viability of HGF exposed to “Tera Harz TA-28”. (**A**) Specimens of various thicknesses (0.5, 1, 2, 4, 6 mm), all post-cured for 20 minutes; (**B**) Proliferation of HGFs exposed to “Tera Harz TA-28”, monitored over time as a fold increase in confluence using life cell imaging. Statistical significance was analyzed using one way Anova, *p* < 0.05*, *p* < 0.01**, *p* < 0.001***, *p* < 0.0001****.

Initially, the effect of Graphy’s aligner resin Tera Harz TA-28 on oral gingival fibroblasts (HGF) was assessed using specimens of different material thicknesses (*n* = 6 for each thickness) that were all post-cured for 20 min after 3D printing. A decrease in cell viability was observed with increasing specimen thickness (0.5 [n.s.], 1 mm [n.s.], 2 mm ***, 4 mm***, 6 mm**) under UV curing performed in accordance with the manufacturer’s specifications. Extending the UV curing time beyond the recommended 20 min did not improve cell viability. Importantly, the reduction in cell viability never exceeded 30%, even at higher thicknesses, indicating that the material meets the non-cytotoxicity criteria outlined in EN ISO 10993-5 (Fig. 3A).Interestingly, there was a little increase in cell viability when comparing specimens of 6 mm thickness to the ones with a thickness of 4 mm.

Live cell imaging over 72 h also showed that the cells proliferated under all conditions (Fig. 3B), with no morphological changes observed and no cells detaching from the well bottom.

**Fig. 4:**
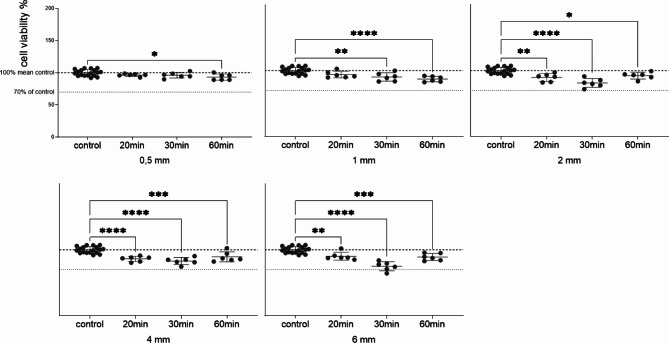
Variation in cell viability assessed following post processing (UV curing) for different durations (20, 30, 60 min). Specimens in media for 12 days and treatment of HGFs with the conditioned media (1:2) for 72 h. Data are presented as individual values (*n* = 6) relative to the control mean (100%), expressed as mean ± SD. Statistical significance was analyzed using one way Anova, *p* < 0.05*, *p* < 0.01**, *p* < 0.001***; *p* < 0.0001****.

Subsequently, the UV curing duration was varied (20, 30 and 60 min). Once again, no reduction in cell viability exceeding 30% was observed (Fig. 4). Cell viability decreased with increasing curing time for all material thicknesses, with specimens of 2, 4 and 6 mm showing the greatest loss in cell viability when cured for 30 min. Curing for 60 min, however, showed a subsequent increase in cell viability.

**Fig. 5:**
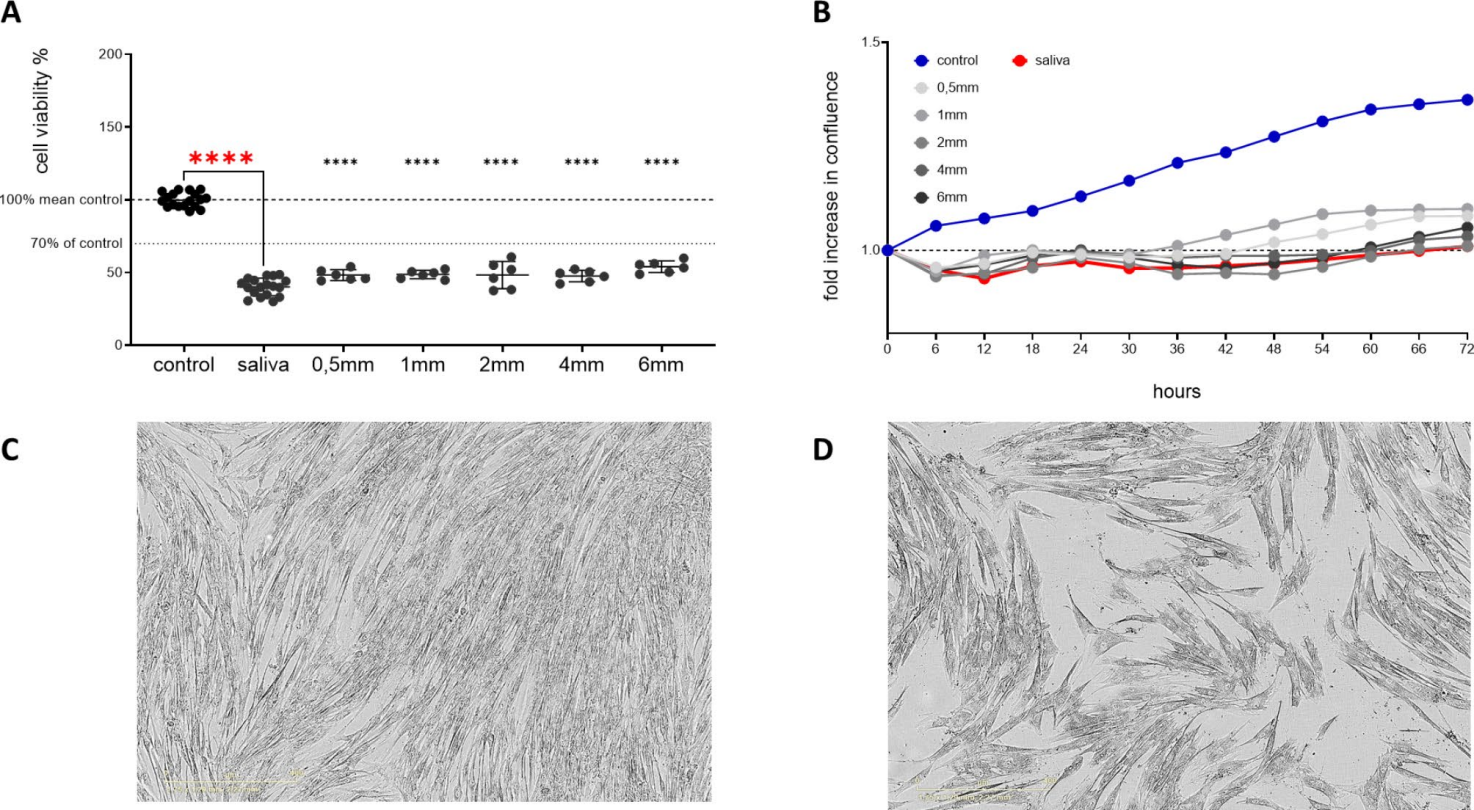
Cell viability of HGFs exposed to “Tera Harz TA-28” in saliva. (**A**) 50% saliva conditioned with specimens of varying thicknesses (0.5, 1, 2, 4, 6 mm), all post cured for 20 min, for 12 days. (**B**) Proliferation of HGFs under “Tera Harz TA-28” with 50% saliva monitored over time as fold increase in confluence by life cell imaging. (**C**) HGFs in media conditioned with specimen of 1 mm thickness. (**D**) HGF in 50% salvia conditioned with specimen of 1 mm thickness.

In a further approach, we investigated the influence of saliva on the potential release of biologically active substances from the aligner material. Therefore, specimens of all different thicknesses (0.5, 1, 2, 4, 6 mm) were post-cured for 20 min and then incubated in saliva for 12 days. After incubating HGFs with the conditioned saliva, a significant drop in cell viability was observed, even when unconditioned saliva was applied (Fig. 5A). Live cell imaging over 72 h further supported this result (Fig. 5B). Microscopic images of cells in media and in salvia (Fig. 5C and D), taken after 72 h, clearly show that HGFs in saliva ceased proliferating, despite appearing to remain viable. Here, it is particularly important to highlight that HGFs more or less stopped proliferating even when unconditioned saliva was applied.

## Discussion

DPAs are anticipated to be a milestone in clear aligner therapy due to their multiple advantages, such as improved precision and fit, compared to traditional clear aligners produced in a thermoforming process^7–11^. One of their key advantages is the ability to design pressure zones by increasing layer thickness in specific areas, which can facilitate the desired tooth movements, reduce side effects and simultaneously improve anchorage^10^. This may reduce the number of necessary attachments for a successful treatment outcome, thereby increasing efficiency by reducing chairside time and simultaneously enhancing aesthetics. To benefit reliably from these benefits, high dimensional accuracy in the 3D printing process could be crucial. Maintaining accurate thickness is of particular importance as it may influence the stiffness, resistance to deformation, elastic modulus, tensile strength as well as stress relaxation of the DPAs^21,22^. This study demonstrated a high level of printing accuracy across different thickness and post-curing time (Fig. 2). This contrasts with previous research reporting that DPAs tend to be thicker than their corresponding STL files^21,23,24^. While the modified material, Tera Harz TA-28, may exhibit differences in properties, such variations are likely influenced by factors such as the choice of printer, printer settings and the post-processing protocols applied^15^.

In addition to customized pressure zones, the variability in the DPAs’ digital design allows orthodontists to produce almost any kind of desired, custom-made removable orthodontic appliance: For instance, posterior bite blocks designed by increasing posterior occlusal layer thickness significantly up to several millimeters can be used to create Class II mechanics similar to the Twin-Block or the Mandibular Advancement Tool (Invisalign^®^, Align Technology, San Joé, CA, USA). Molar intrusion in patients with anterior open bite can be facilitated as well.

However, from our perspective, these benefits come with the risk of exposing patients to cytotoxic monomers as a longer UV curing duration might be needed for the UV light to fully polymerize the whole volume in areas of higher material thickness. Insufficient or incomplete curing of the resin may increase the potential for adverse side effects.

During active treatment, strict adherence to the essential wearing time of 22 + hours daily is crucial for successful treatment outcome. Consequently, the material remains in almost continuous contact with the patient’s oral environment. Given the typical 7- to 14-day protocols, the initial high release rate of a newly worn DPA is consistently maintained throughout the entire active treatment period^20^. Both factors increase the importance of the material’s biocompatibility significantly as oral mucosa irritation has already been detected in some patients^19^. Although the rapidly increasing interest in DPAs made from Graphy’s resins has resulted in numerous research studies, most of these focus on the DPAs’ mechanical properties, accuracy and biomechanical behavior^10–15,21,23,25^. Only a few studies address the biocompatibility and potential risk of cytotoxicity of Tera Harz TC-85^17–20^. Pratsinis et al. demonstrated that the eluates of DPAs made from Tera Harz TC-85 do not have a cytotoxic effect on human gingival fibroblasts, nor do they induce the proliferation of estrogen-sensitive MCF-7 cells. However, their study did not vary layer thickness, post-curing duration, or the 3D printer used^18^. Willi et al. observed considerable variability in UDMA leaching from DPAs made from Tera Harz TC-85, which could pose a potential health risk to patients^20^. Campobasso et al. examined the impact of varying post-curing devices and durations on biocompatibility. They reported that using the Tera Harz Cure (Graphy Inc, Seoul, South Korea) for 14 min in a nitrogen atmosphere, as recommended for Tera Harz TC-85 by the manufacturer, resulted in high biocompatibility with no evidence of cytotoxicity. In contrast, DPAs post-cured with the Form Cure (FormLabs Inc, Somerville, USA) for 60 min (30 min per side) exhibited moderate cytotoxicity^17^. Similarly, Iodice et al. investigated the effects of different curing durations using the Tera Harz Cure (Graphy Inc, Seoul, South Korea) and observed a negative correlation between curing time and fibroblast proliferation, indicating increased cytotoxicity with prolonged curing durations^19^. This is in line with our results for Tera Harz TA-28 when considering an identical thickness of 0.5 mm, although only the increase in curing time to 60 min showed statistical significance. The observed negative correlation could potentially be explained by the effects of prolonged UV curing on the material’s characteristics such as increased roughness, porosity, hardness, morphology and residual stress. Previous research has suggested that smooth surfaces might be better in facilitaing cell growth, and that the exposure to higher temperatures during a longer curing process could have an impact on the surface properties^19^. Recent findings reported no significant effects of heat exposure during post-processing on the DPAs’ mechanical properties^26^. However, corresponding effects on cytotoxicity have not yet been assessed.

This study evaluates the biocompatibility of DPAs made from Graphy’s new aligner resin, Tera Harz TA-28, as well as assesses the effects of different material thicknesses on cell viability. These findings will contribute to a deeper understanding of the material’s properties and its potential clinical applications. Until now, it was unclear whether such design adaptations would require significant alterations in the post-curing process to still guarantee biological safe use in patients. When following the company’s curing guidelines and curing the specimens for 20 min each, a slight loss of cell vitality is detected with increasing thickness. To further evaluate the interplay between DPAs’ thickness, curing time, and the resulting cytotoxicity in more detail, the effects of eluates from specimens of different thicknesses, cured for either 20, 30, or 60 min, on HGFs were analyzed. For specimens with 0.5 mm and 1 mm thickness, cell viability decreased with increasing curing time. However, specimens with a thickness of 2, 4, or 6 mm showed the greatest loss in cell viability at a curing time of 30 min, with viability increasing again at 60 min (Fig. 3). Although the decrease in cell viability for all combinations was less than 30% and therefore not considered cytotoxic according to the ISO 10993-5 standard, our findings challenge Graphy’s recent recommendation to extend UV curing by an additional 10 min for DPAs made from TC-85 that are thicker than 0.7 mm^27^. However, it needs to be considered that we did not flip the specimens after the standard of 20 min as suggested in this new recommendation for thicker DPAs. If any prolongation of UV curing is considered, it should be significantly extended to 60 min. Nevertheless, no significant change was observed compared to the standard 20-minute UV curing duration. Consequently, to optimize time and enhance productivity in daily practice, we recommend not to increase UV curing duration for layer thicknesses of up to 6 mm. The present findings highlight that saliva exposure impacts cell viability, as observed through the significant reduction in cell proliferation during saliva incubation. This emphasizes the necessity of considering intraoral factors such as salivary enzymes, pH variations and microbial interactions, which may alter material biocompatibility.

Similar to previous research, one limitation of the current study, being in-vitro, is the inability to fully simulate all potential intraoral influences including chewing forces and bruxism which may damage the DPAs surface exposing inner layers that might not be fully cured, pH, temperature fluctuations, microbial activity as well as enzymatic reactions (e.g. esterases)^18–20^. Nevertheless, this provides a good and, in our opinion, also reliable indication of whether an adjustment in curing duration is needed for DPAs with increased layer thickness to ensure biocompatibility. However, the standard of 20 min seems perfectly suitable.

## Conclusions

The introduction of DPAs using Graphy’s aligner resins marks a significant innovation over traditional thermoformed aligners. The current study confirmed the high accuracy of the production method, as all specimens achieved their target dimensions irrespective of the UV curing time. The standard 20-minute UV curing protocol was sufficient to ensure biocompatibility and, thus, patient safety for material thicknesses up to 6 mm.

## Data Availability

All datasets are available upon request from the corresponding author.
